# Clinical performance of metagenomic next-generation sequencing for distinction and diagnosis of *Mucorales* infection and colonization

**DOI:** 10.3389/fcimb.2025.1631960

**Published:** 2025-09-18

**Authors:** Xiaoli Zhou, Chenxi Yang, Xin Liu, Jiaqiang Wang, Yanqiao Li, Lingai Pan, Shengkun Peng, Hua Yu, Xiren Deng

**Affiliations:** ^1^ Department of Laboratory Medicine, Sichuan Provincial People’s Hospital, University of Electronic Science and Technology of China, Chengdu, China; ^2^ Department of Dermatology, Sichuan Provincial People’s Hospital, School of Medicine, University of Electronic Science and Technology of China, Chengdu, China; ^3^ Department of Critical Care Medicine, Sichuan Provincial People’s Hospital, University of Electronic Science and Technology of China, Chengdu, China; ^4^ Department of Radiology, Sichuan Provincial People’s Hospital, University of Electronic Science and Technology of China, Chengdu, China

**Keywords:** mucormycosis, *Mucorales*, diagnosis, metagenomic next-generation sequencing, optimal threshold value

## Abstract

Mucormycosis is a lethal fungal infection disease with high mortality rate. However, investigations assessing the value of metagenomic next-generation sequencing (mNGS) for distinguishing *Mucorales* infection from colonization are currently insufficient. A retrospective analysis of clinical date from 71 patients at Sichuan Provincial People’s Hospital from September 2021 to September 2024 was conducted. The performance of mNGS in distinguishing *Mucorales* infection from colonization, along with the differences in patients’ characteristics, imaging characteristics, antimicrobial adjustment, and microbiota, were examined. Among the 71 patients, 51 were identified as *Mucorales* infection group (3 proven and 48 probable cases), and 20 were colonization group (possible cases). Receiver operating characteristic (ROC) curve for mNGS indicated an area under the curve of 0.7662 (95%CI: 0.6564-0.8759), with an optimal threshold value of 51 for discriminating *Mucorales* infection from colonization. The infection group exhibited a higher proportion of antimicrobial adjustments compared to the colonization group (64.71% *vs*. 35.00%, *P* < 0.05), with antifungal agent changed being more dominant (43.14% *vs*. 10.00%, *P* < 0.01). *Mucorales* RPTM value, length of hospital stays, hsCRP, immunocompromised, malignant blood tumor, and antifungal changed were significantly positively correlated with *Mucorales* infection. *Rhizomucor pusillus* showed significant differences between the two groups. The abundance of *Torque teno virus* significantly increased in the infection group, whereas the colonization group exhibited higher abundance of *Rhizomucor delemar*. mNGS is a valuable tool for differentiating colonization from infection of *Mucorales*. Malignant blood tumor, immunocompromised, length of hospital stays and hsCRP were significant different indicators between patients with *Mucorales* infection from colonization.

## 1 Introduction

Mucormycosis, a lethal and opportunistic infection disease caused by fungi of the order *Mucorales*, aggressively invades human blood, organs, and tissues (Donnelly et al., 2020; Panda et al., 2024; Pappas et al., 2021). The *Mucorales* order comprises 55 genera and 261 species, with 38 recognized as pathogenic to humans. *Rhizopus arrhizus* is the most prevalent pathogenic genus globally, followed by *Mucor* and *Rhizomucor*, while *Apophysomyces* and *Cunninghamella* are less frequently implicated. These fungi are ubiquitous in the environment and exhibit a high propensity for colonizing the human respiratory tract (Liang et al., 2024; Roden et al., 2005). Although *Mucorales* colonization does not immediately provoke disease, it serves as a prerequisite for chronic and allergic mycoses, as well as localized airway infections in invasive fungal diseases. The diagnosis relies on histopathological analysis, conventional microbiological testing (CMT), and imaging, with histopathology or culture considered as the “gold standard” for diagnosis of mucormycosis (Donnelly et al., 2020; Pappas et al., 2021). Histopathological analysis of sterile specimens was critical for confirmation, but there exist difficulties in sampling (Hammer et al., 2018). For CMT, including culture and direct microscopic examination of specimens, also faces limitations in timely diagnosing mucormycosis (Skiada et al., 2018; Wang et al., 2024). While other microbiological testing (OMT) like the galactomannan (GM) antigen testing and (1–3)-β-D-glucan (G) testing have difficulties with accuracy (Lass-Flörl et al., 2021; Lmoth et al., 2021). On account of nonspecific symptoms and signs, early mucormycosis identification is still a challenge in clinic. Definitive diagnosis of mucormycosis, particularly distinguishing between colonization and active infection, remains a significant clinical hurdle (Sipsas et al., 2018; Skiada et al., 2018). However, there remains a paucity of studies focused on differentiating *Mucorales* infection from colonization.

mNGS is a unbiased sequencing of all nucleic acids (DNA/RNA) in clinical samples (blood, cerebrospinal fluid, respiratory secretions, etc.), and the identification of pathogens (bacteria, viruses, fungi, parasites) through bioinformatics comparison. It does not require pre-assumption of pathogens and is suitable for detecting unknown infections, mixed infections, or rare pathogens (Gu et al., 2019). While, nucleic acid testing by PCR for single agents to multiplexed PCR testing using syndromic panels generally include the most common pathogens associated with a defined clinical syndrome (Chiu and Miller, 2019). The application of metagenomic next-generation sequencing (mNGS) has gained prominence in the clinical diagnosis of infectious diseases, particularly when empirical anti-infective therapies prove ineffective or when CMT fails to identify the etiology. Compared to CMT, mNGS demonstrates superior diagnostic performance for invasive fungal infections, and multiple studies highlight its ability to detect fungal pathogens undiagnosable by traditional methods (Jia et al., 2023; Jiang et al., 2024; Liu et al., 2021; Wang et al., 2024; Zhang et al., 2024, 2024).

Furthermore, the utility of mNGS in differentiating fungal colonization from infection have been explored, primarily by establishing thresholds for pathogen-specific read counts. For instance, Liu et al. demonstrated that bronchoalveolar lavage fluid (BALF) mNGS could distinguish *Pneumocystis jirovecii* colonization from infection with an area under the curve (AUC) of 0.973, identifying an optimal threshold of 14 reads (Liu et al., 2021). Jia et al. reported a species-specific read number (SSRN) cut-off of 2.5 for diagnosing invasive pulmonary aspergillosis (IPA) versus non-IPA, with distinct thresholds of 1 and 4.5 for immunocompromised and diabetic IPA patients, respectively (Jia et al., 2023). Similarly, the study of Jiang et al. discovered an optimal mNGS RPTM (reads per ten million) cut-off value of 23 for discriminating between *Aspergillus* infection and colonization (Jiang et al., 2024). Despite these advancements, critical gaps persist in understanding the clinical characteristics of patients and the microbial compositional differences between those with *Mucorales* colonization and infection.

In this study, we evaluated the efficacy of mNGS, culture and OMT in distinguishing *Mucorales* infection from colonization. Furthermore, we delineated variations in antimicrobial management strategies, clinical indicators, and shifts in pulmonary microbial composition between these patient groups.

## 2 Materials and methods

### 2.1 Study design and participants

This retrospective study included 71 patients with mucormycosis hospitalized at the Sichuan Provincial People’s Hospital from September 2021 to September 2024. The corresponding medical records were reviewed, and the clinical data analyzed including demographic characteristics, type of underlying disease, diagnosis, clinical course, treatment, and outcome.

BALF, blood, SCF and tissue were used for pathogen identification through CMT, including culture for bacteria (blood agar plates, Chocolate, and MacConkey) and fungi (Sabouraud agar plates), and OMT methods, including 1-3-β-D-glucan (G) test (Fungi (1,3)-β-D-glucan assay kit, Gold Mountainriver Tech Development Co.,LTD, Beijing, China), galactomannan (GM) test (Galactomannan test kit, Dana Biotechnology Co.,LTD, Tianjing, China) and smear microscopy for fungi (KOH or Phenol cotton orchid stain), aiming to provide a methodological assessment.

### 2.2 Criteria for *Mucorales* infection diagnosis

In this study, the diagnoses of invasive *Mucorales* infection were classified into proven, probable and possible cases based on the guidelines performed by the European Organization for Research and Treatment of Cancer/Mycoses Study Group Education and Research Consortium (EORTC/MSGERC) (Donnelly et al., 2020). Proven cases required adhere to host factors, clinical signs or symptoms, and positive results from microbiological and/or histopathological examination. The microbiological criteria include microscopic examination and *Mucorales* recovered by culture from specimens obtained through aseptic procedures from normally sterile, clinically, or radiologically abnormal sites consistent with an infectious disease process. For histopathology, needle aspiration or biopsy revealed hyphae, and accompanied by evidence of associated tissue damage. Probable *Mucorales* infection is definite as the presence of at least one host factor, a clinical feature and mycologic evidence. Alternatively, a joint diagnosis by imaging experts and clinical doctors of the hospital was needed in case of mycological evidence has not been found or detection of the same *Mucorales* pathogen through mNGS on more than two occasions. Possible cases meet the criteria of with a host factor and a clinical feature of *Mucorales* infection, but not mycologic criteria. Proven and probable cases were classified into *Mucorales* infection group, and possible cases were classified into *Mucorales* colonization group (Donnelly et al., 2020; Feys et al., 2022; Jiang et al., 2024). Two experienced physicians made clinical diagnoses; when they gave different results, another senior physician made a judgement. Therefore, patients with host factors, obvious clinical signs or symptoms but without positive mycological results were classified as *Mucorales* infection, as well the cases were considered as colonization when *Mucorales* was identified but without a final diagnoses of *Mucorales* infection (Donnelly et al., 2020; Feys et al., 2022; Jiang et al., 2024).

### 2.3 Sample collation and mNGS detection

Clinical samples, including blood, BALF, CSF, pus, pleural fluids, and tissue, were collected using aseptic techniques when clinicians suspects a pathogenic microorganism infection but has not yet found etiological evidence. And chemical DNA or RNA stabilizers were used to minimize the possibility of nucleic acid degradation at the time of sample collection. The detailed methods regarding the wet lab and bioinformatics had been described previously (Zhou et al., 2022). Briefly, nucleic acids were extracted using the TIANamp Micro DNA Kit (DP316, TIANGEN BIOTECH, Beijing, China). The extracted DNA underwent fragmentation, end repair, adapter ligation and sequencing. Quality assessment was performed using the Agilent 2100 system and sequencing was conducted on the MGISEQ-2000 platform (BGI Genomics Co.,Ltd., Shenzhen, China).

### 2.4 ROC curve construction

The ROC curve is constructed based on the *Mucorales* RPTM values detected by mNGS. The RPTM value reflects the load of *Mucorales* in the sample and is a core indicator for distinguishing infection from colonization. According to guidelines performed by the EORTC/MSGERC, patients were divided into infection group and colonization group. By calculating the sensitivity and specificity at different RPTM thresholds, ROC curves were plotted, and the Youden index (sensitivity+specificity -1) was used to determine the optimal cut-off value.

### 2.5 Statistical analysis

The data were analyzed by descriptive statistics. The chi-square test was applied to the categorical variables. A student t-test was used for continuous variables. *P*-value less than 0.05 was considered statistically significant. All statistical analyses were performed using GraphPad Prism (Version 8.0.2, GraphPad Software Inc) and SPSS (Version 25, IBM Corp). The diagnostic performance of mNGS was evaluated using the area under the curve of receiver operating characteristic (ROC), where the best cut-off value was obtained. The sensitivity and specificity of the detection method were analyzed as reference (Blauwkamp et al., 2019). The correlation analysis was conducted in R by the corrplot package. The alpha diversity index was calculated based on Shannon and Simpson indexes. Beta-diversity was visualized using principal coordinate analysis (PCoA), and an ANOSIM test was performed in R with the Vegan package. The stacked bar plot of the community composition was visualized in R using the ggplot2 package. Linear discriminant analysis (LDA) effect size (LEfSe) was utilized by R with microeco package to identify significantly different species among the groups, with thresholds of log_10_ LDA Score ≥ 2 and *P* value ≤ 0.05.

## 3 Results

### 3.1 Baseline characteristics and sample classification

Totally, 71 patients were included and diagnosed as proven (n = 3), probable (n = 48) and possible (n = 20) mucormycosis. Among them, 51 were identified as *Mucorales* infection, and 20 were colonization group.

According to **Table 1**, the median age at diagnosis was 57 years old (ranged from 9 to 103), and most were males (70.42%, n = 50). The significant differences in *Mucorales* infection and colonization groups were observed including malignant blood tumor (n =15 *vs*. n = 1, *P* = 0.0294), longer length of hospital stays (LOHS) (29.57 *vs*. 19.45 days, *P* = 0.0494), immunocompromised (n = 26 *vs*. n = 4, *P* = 0.0311), and hsCRP level (127.25 *vs*. 54.16 ug/mL, *P* = 0.0014).

**Table 1:** General demographic and clinical characteristics of the patients with *Mucorales* infection and colonization.

Characteristics ^a^	All patients (n = 71)	Mucorales infection (n = 51)	Mucorales colonization (n = 20)	P-value ^b^
Age, mean ± SD (Year)	57.39 ± 18.50	55.61 ± 20.53	61.95 ± 18.24	0.1560
Gender (Male)	50 (70.42%)	35 (68.63%)	15 (75%)	0.7742
Underlying condition				
Diabetes mellitus	29	21	8	0.9290
Malignant blood tumor	16	15	1	0.0294^*^
Transplant	5	3	2	0.6161
Hypertension	20	13	7	0.5583
Liver disease	8	7	1	0.4267
Renal disease	20	13	7	0.5583
Smoking	11	6	5	0.2717
COPD	9	7	2	0.7243
Symptoms				
Fever	17	12	5	0.8979
Cough	24	18	6	0.7840
Expectoration	16	13	3	0.3744
Chest distress	6	3	3	0.3404
Chest pain	2	1	1	>0.9999
Hemoptysis	4	2	2	0.5713
Immunocompromised	30	26	4	0.0311^*^
LOHS(day)	26.72 ± 28.26	29.57 ± 23.29	19.45 ± 18.61	0.0494^*^
Types of mucormycosis				
Pulmonary mucormycosis	35	35	0	<0.0001^*^
Rhino-orbital-cerebral mucormycosis	6	6	0	0.1747
Disseminated mucormycosis	6	6	0	0.1747
Clinical test				
hsCRP (ug/mL)	107.12 ± 83.56	127.25 ± 83.27	54.16 ± 58.41	0.0011^*^
PCT (ng/mL)	8.88 ± 20.03	8.22 ± 18.07	10.83 ± 25.58	0.8801
WBC (×10^9^/L)	9.97 ± 8.22	9.47 ± 6.78	11.32 ± 11.33	0.9561
RBC (×10^12^/L)	3.27 ± 1.01	3.20 ± 0.96	3.44 ± 1.15	0.3845
NEUT (×10^9^/L)	7.57 ± 5.93	7.86 ± 6.34	6.78 ± 4.69	0.6205
Lym count (×10^9^/L)	0.91 ± 0.90	0.78 ± 0.59	1.26 ± 1.40	0.2655
PLT (×10^9^/L)	168.44 ± 133.82	166.01 ± 135.24	174.95 ± 133.36	0.8571
NEUT %	71.26 ± 25.94	70.16 ± 27.61	74.21 ± 21.18	0.9295
Lym %	16.98 ± 21.44	18.70 ± 24.40	12.37 ± 8.78	0.9817
Hb (g/L)	96.61 ± 27.03	95.40 ± 26.42	99.84 ± 28.53	0.6442
Cr (umol/L)	162.00 ± 200.38	150.63 ± 192.91	192.54 ± 221.79	0.2598
TBIL (umol/L)	32.15 ± 55.95	29.68 ± 57.79	38.75 ± 51.58	0.0891
ALT (U/L)	107.59 ± 493.37	31.59 ± 34.30	311.58 ± 932.48	0.8777
AST (U/L)	379.86 ± 2396.20	43.24 ± 41.14	1283.42 ± 4563.18	0.7359
LDH (U/L)	685.89 ± 1646.37	490.23 ± 598.77	1211.11 ± 2999.14	0.7708
ALP (U/L)	122.31 ± 77.80	127.19 ± 81.50	109.21 ± 67.09	0.2543
GGT (U/L)	90.04 ± 141.77	82.38 ± 118.54	110.58 ± 193.39	0.9661
IL-2 (pg/ml)	2.41 ± 1.40	2.34 ± 1.53	2.61 ± 1.04	0.6536
IL-4 (pg/ml)	2.87 ± 4.00	2.23 ± 1.64	4.22 ± 6.70	0.2256
IL-6 (pg/ml)	1254.23 ± 4421.44	724.41 ± 1546.48	2747.39 ± 8345.88	0.1385
IL-10 (pg/ml)	76.05 ± 188.68	90.58 ± 221.11	44.09 ± 83.03	0.6170
TNF-α (pg/ml)	2.65 ± 2.30	2.50 ± 2.63	2.96 ± 1.36	0.1636
INF-γ (pg/ml)	4.24 ± 9.19	4.94 ± 10.88	2.78 ± 3.86	03829
CD3+%	70.02 ± 17.54	70.22 ± 18.37	69.44 ± 15.69	0.6907
CD3+# (/ul)	656.49 ± 885.16	697.5 ± 999.47	540.91 ± 442.35	0.6202
CD3+CD4+%	36.18 ± 15.22	35.56 ± 15.84	38.00 ± 13.82	0.6512
CD3+CD4+# (/ul)	299.01 ± 282.41	294.75 ± 297.51	311 ± 247.50	0.6518
CD3+CD8+%	32.33 ± 16.00	32.78 ± 17.62	31.02 ± 10.53	0.7571
CD3+CD8+# (/ul)	340.73 ± 651.84	380.67 ± 748.54	228.18 ± 205.59	0.9157
CD3+CD4-CD8-%	3.42 ± 6.52	3.76 ± 7.44	2.43 ± 2.38	0.5679
CD3+CD4-CD8-# (/ul)	22.16 ± 62.33	25.15 ± 71.95	13.73 ± 17.07	0.7187
CD3+CD4+CD8+%	1.93 ± 1.87	1.60 ± 1.33	2.88 ± 2.80	0.1088
CD3+CD4+CD8+# (/ul)	12.55 ± 21.42	10.65 ± 19.27	17.91 ± 26.92	0.4827
CD3-CD19+%	14.19 ± 10.11	13.57 ± 11.24	16.66 ± 2.46	0.3168
CD3-CD19+# (/ul)	87.53 ± 58.50	92.89 ± 57.79	68.75 ± 65.68	0.6021
CD3-CD16+CD56+%	14.22 ± 12.97	12.36 ± 12.91	21.18 ± 12.23	0.1775
CD3-CD16+CD56+# (/ul)	88.23 ± 63.61	93.14 ± 55.52	72.25 ± 93.83	0.7034
CD3+CD16+CD56+%	3.83 ± 4.43	3.29 ± 4.21	5.87 ± 5.28	0.5536
CD3+CD16+CD56+# (/ul)	34.80 ± 74.85	38.82 ± 84.33	21.75 ± 33.63	0.5582
CT findings				
Nodules	31	23	8	0.7932
Consolidation	18	16	2	0.0752
Ground glass shadow	30	24	6	0.2857
Tree in bud	5	3	2	0.6161
Cavities	8	6	2	>0.9999
Patchy shadow	43	32	11	0.5963
Pulmonary emphysema	6	4	2	>0.9999
Pleural effusion	8	4	4	0.2090

aCOPD, chronic obstructive pulmonary disease; LOHS, length of hospital stays; CRP, C-reactive protein; PCT, Procalcitonin; WBC, white blood cell; RBC, red blood cell; NEUT, neutrophil; Lym, lymphocyte; PLT, platelet; Hb, hemoglobin; Cr, creatinine; TBIL, total bilirubin; ALT, alanine aminotransferase; AST, aspartate aminotransferase; LDH, lactate dehydrogenase; ALP, Alkaline phosphatase; GGT, γ-glutamyl transpeptidase. bAnalysis of significant differences between baseline data of Mucorales infection and colonization patients. * Indicated that the P-value < 0.05.

**Figure 1:**
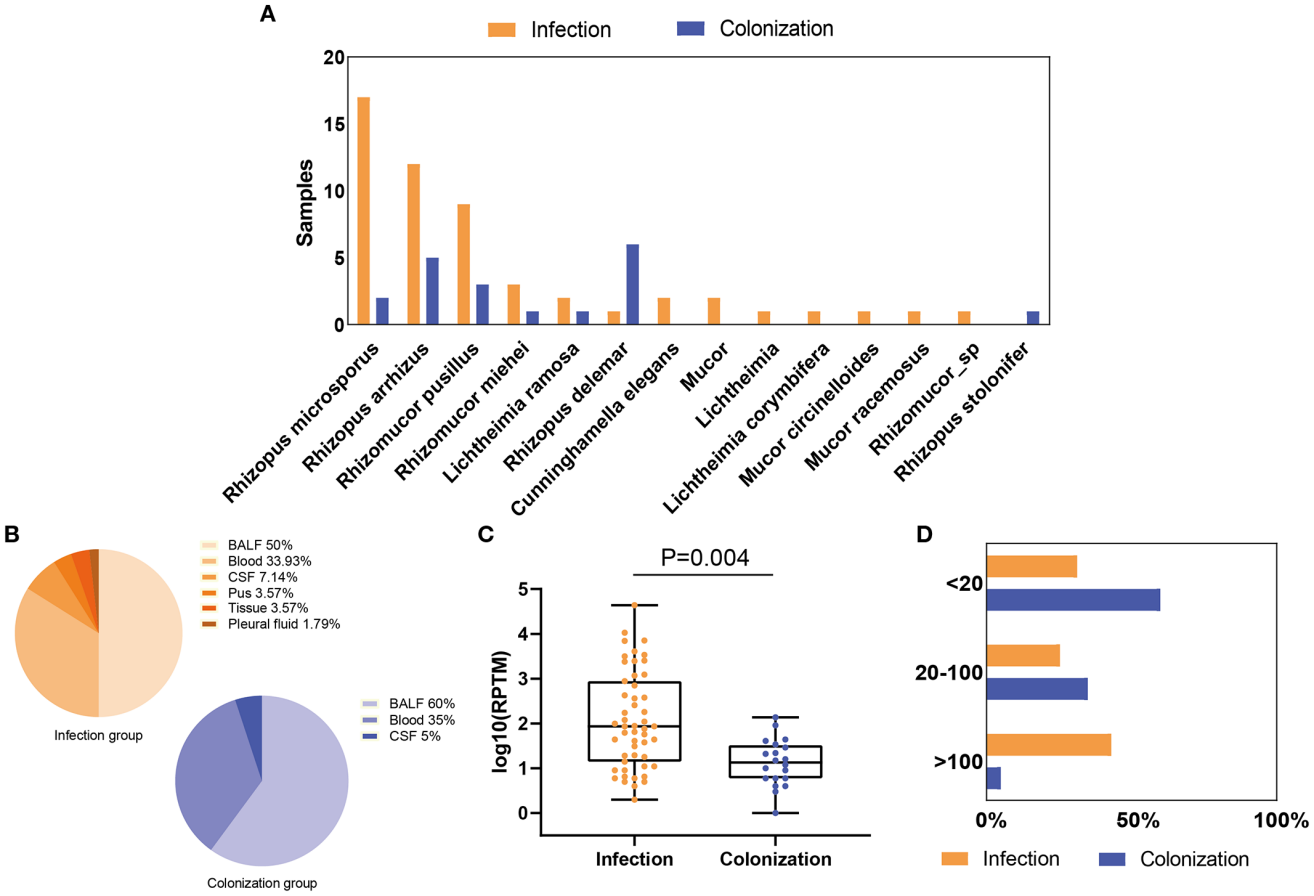
Distribution and abundance of *Mucorales* species in patients with *Mucorales* infection and colonization. **(A)** Comparison of *Mucorales* species in patients with *Mucorales* infection and colonization. **(B)** Distribution of sample types in patients with *Mucorales* infection and colonization. **(C)** Differences in mNGS RPTM for *Mucorales* in patients with *Mucorales* infection and colonization. **(D)** Proportion of patients with different mNGS *Mucorales* reads in the infection and colonization groups.

In the *Mucorales* infection and colonization groups, 13 and 7 *Mucorales* species were identified by mNGS, respectively (**Supplementary Table 1**). In *Mucorales* infection group, *Rhizopus microsporus* (33.33%, 17/51) was the most common species, followed by *Rhizopus arrhizus* (23.53%, 12/51) and *Rhizomucor pusillus* (17.65%, 9/51). Three patients were found to be co-infected with *Rhizopus* and *Mucor*, including two patients co-infected with *Rhizopus microsporus* and *Mucor*, and one patient co-infected with *Rhizopus microsporus* and *Mucor racemosus*. In *Mucorales* colonization group, *Rhizopus delemar* (30%, 6/20), *Rhizopus arrhizus* (25%, 5/20) and *Rhizomucor pusillus* (15%, 3/20) were the top three of the *Mucorales* species detected (**Figure 1A**). The most frequent sample type observed was BALF, followed by blood (**Figure 1B**; **Supplementary Table 1**). The *Mucorales* load was significantly higher in the infection group compared with colonization group, with a median mNGS read number of 1.82 ± 0.98 *vs*. 1.12 ± 0.53 (*P* = 0.004) (**Figure 1C**). Besides, over 68% of patients in the infection group had an RPTM value larger than 20, while the percentage of colonization group less than 20 was 60%. (**Figure 1D**).

### 3.2 Diagnostic efficacy of mNGS for *Mucorales* infection and colonization

**Figure 2:**
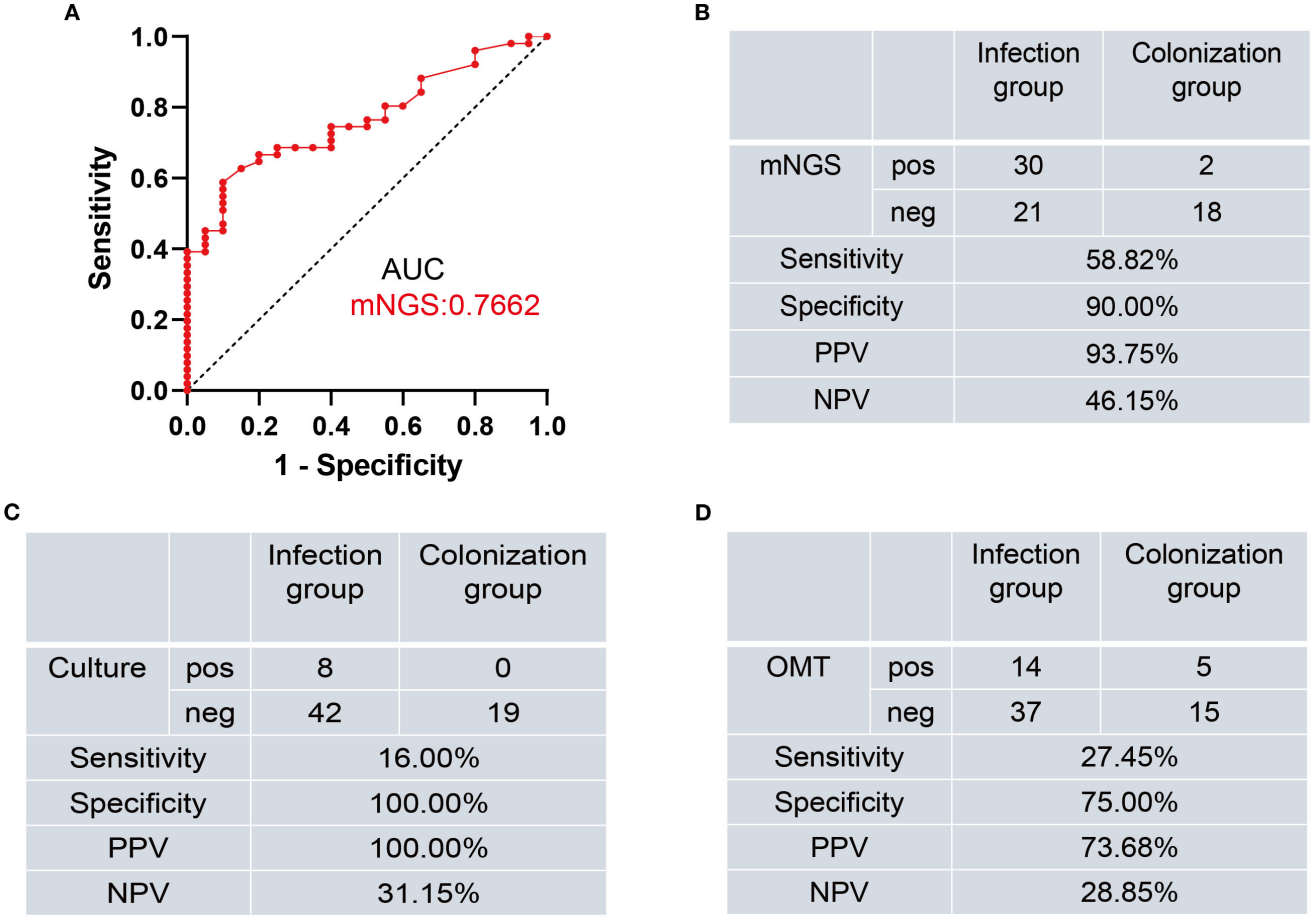
Diagnostic performance of mNGS, Culture, and OMT methods for distinguishing *Mucorales* infection from colonization. **(A)** ROC curve of mNGS for discrimination between *Mucorales* infection and colonization. **(B–D)** Diagnostic performance of mNGS **(B)**, Culture **(C)**, and OMT **(D)** methods for differentiating between *Mucorales* infection and colonization. AUC, area under curve; PPV, positive predictive value; NPV, negative predictive value; pos, positive; neg, negative.

To calculate the cut-off that best discriminated between patients with *Mucorales* infection from colonization, we created a ROC curve using the *Mucorales* RPTM of mNGS from the patients. The calculated area under curve was 0.7662 (95% CI: 0.6564-0.8759), with the optimal cut-off value was determined to be 51 (**Figure 2A**).

Subsequently, we evaluated the diagnostic efficacy of mNGS, culture and OMT in distinguishing infection from colonization (**Supplementary Table 1**). When using RPTM ≥ 51 as the threshold criterion for *Mucorales* infection and colonization, the sensitivity of mNGS was 58.82%, which was significantly higher than culture (16.00%, *P* < 0.001) and OMT (27.45%, *P* = 0.0025). For specificity, there was no significant difference between mNGS and culture (90.00% *vs*. 100.00%, *P* = 0.4872), nor between mNGS and OMT (90.00% *vs*. 75.00%, *P* = 0.4075). While the specificity of culture was significantly higher than that of OMT (*P* = 0.0471) (**Figures 2B–D**).

### 3.3 Diagnostic value of imaging for *Mucorales* infection and colonization

**Figure 3:**
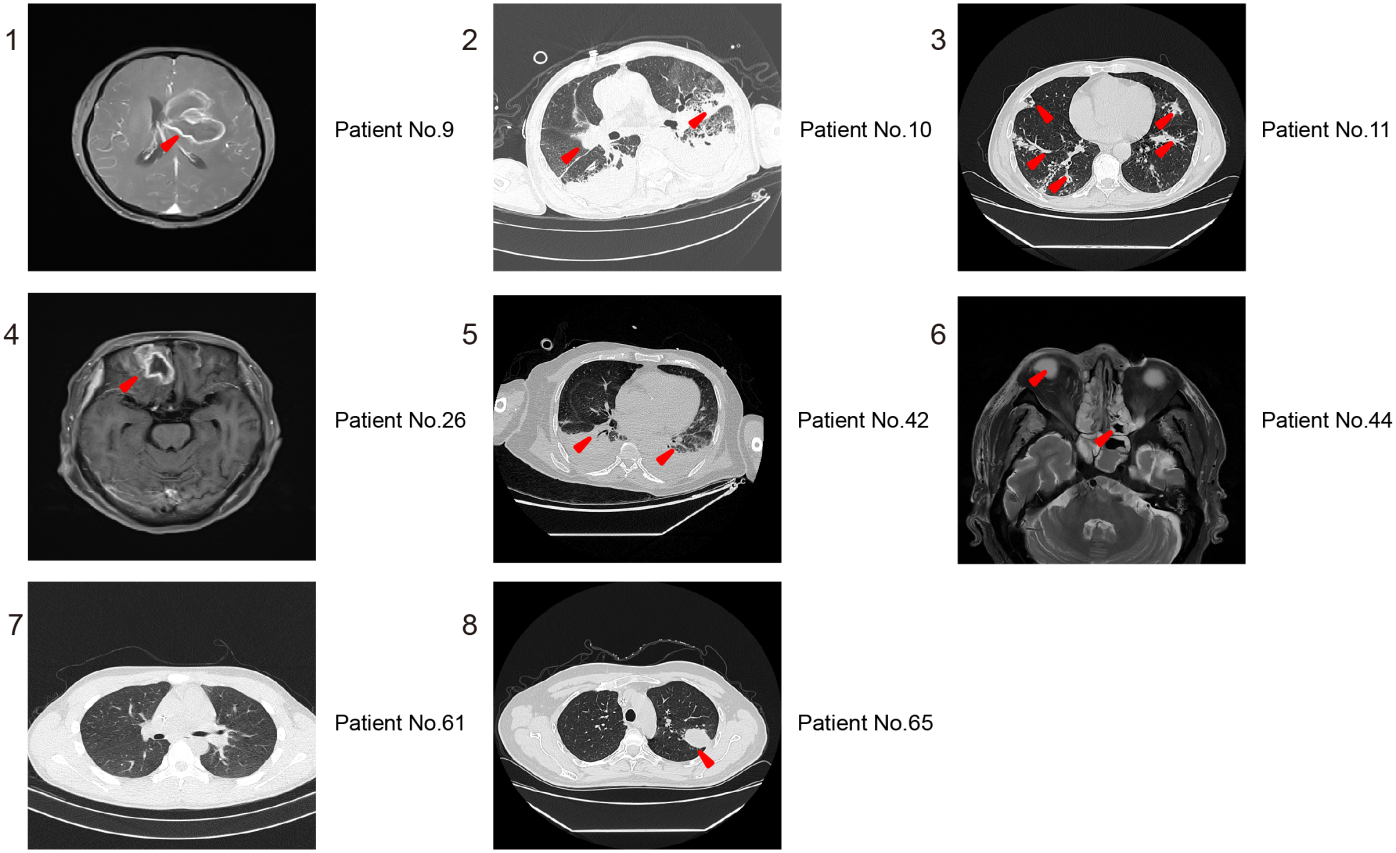
Representative imaging results for distinguishing *Mucorales* infection from colonization. 1,4, and 6 are MRI of Brain; 2, 3, 5, 7, and 8 are CT of lung. The red arrowheads showed the abnormal lesions.

To evaluate the value of imaging in diagnosing *Mucorales* infection, we reviewed the imaging results of all cases. As shown in **Figure 3**, the Brain MRI of patient No.9 showed abnormal lesions, but it can’t indicate which pathogen caused it. Patient No.10 displayed a mixed infection, but cannot be distinguished. Patient No.61 presented no abnormalities. The remaining patients of No. 11, No. 26, No. 42, No. 44, No.65 are all not that obvious for *Mucorales* infection diagnosis. Altogether, it is difficult to determine whether the detected abnormalities are caused by *Mucorales*. Therefore, it is of great significance to combine other laboratory tests for the diagnosis of *Mucorales* infection.

### 3.4 Impacts of mNGS on antimicrobial usage of *Mucorales* infection patients

**Figure 4:**
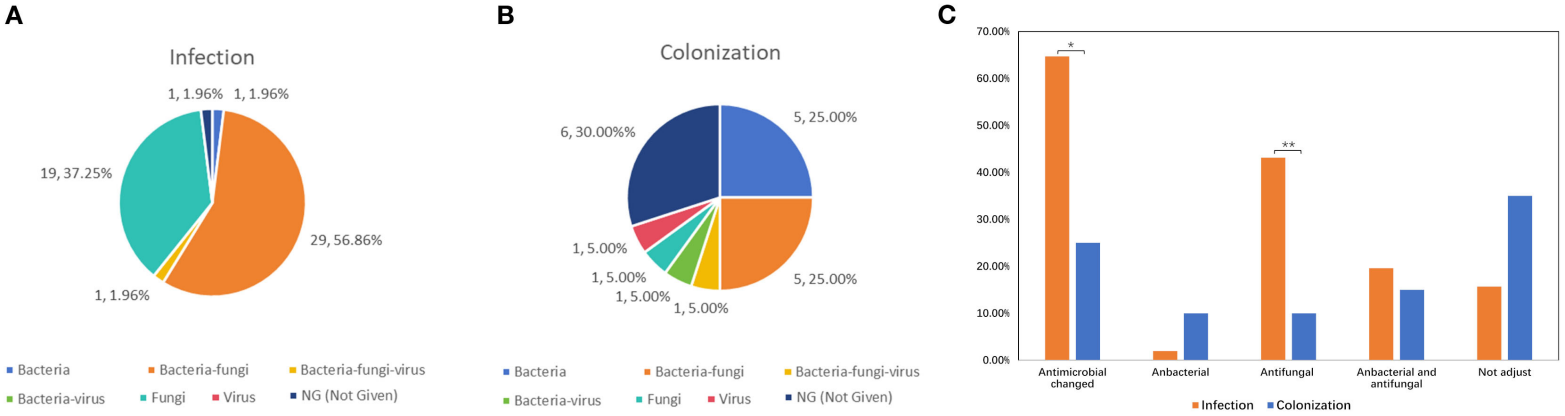
Impacts of mNGS on antimicrobial adjustment in patients with *Mucorales* infection and colonization. **(A, B)** The infection types of patients with *Mucorales* infection **(A)** and colonization **(B)**. **(C)** Variations in antimicrobial regimens of antibacterial and antifungal agent before and after mNGS detection. *P < 0.05, **P < 0.01.

The incidence of bacterial and fungal co-infection was higher in both infection group (56.86%, 29/51) and colonization group (25.00%, 5/20) (**Figures 4A, B**). To explore the influence of mNGS results on antimicrobial usage, we analyzed variations in antimicrobial regimens of antibacterial and antifungal agent before and after mNGS detection. As results in **Figure 4C**, the antimicrobial regimens were adjusted in 33 out of 51 (64.71%) samples from patients with *Mucorales* infection, which was significantly higher than that in *Mucorales* colonization (35.00%, *P* < 0.05). Among the 33 samples, 22 samples had their antifungal agent changed, 10 cases had both antibacterial and antifungal agents adjusted, while one case had their antibacterial changed. The percentage of patients requiring antifungal agent adjusted was significantly higher in *Mucorales* infection group compared to colonization group (43.14% *vs*. 10.00%, *P* < 0.01). Moreover, among 22 patients of infection group who received antifungal treatment, 15 (68.18%) showed improvements, 2 (9.09%) died, and 5 (22.73%) were discharged voluntarily. And, among 10 patients who received both antibacterial and antifungal treatment, 7 (70.00%) have improved, 1 (10.00%) died, and 2 (20.00%) were discharged voluntarily.

### 3.5 Correlations between the characteristics and *Mucorales* infection

**Figure 5:**
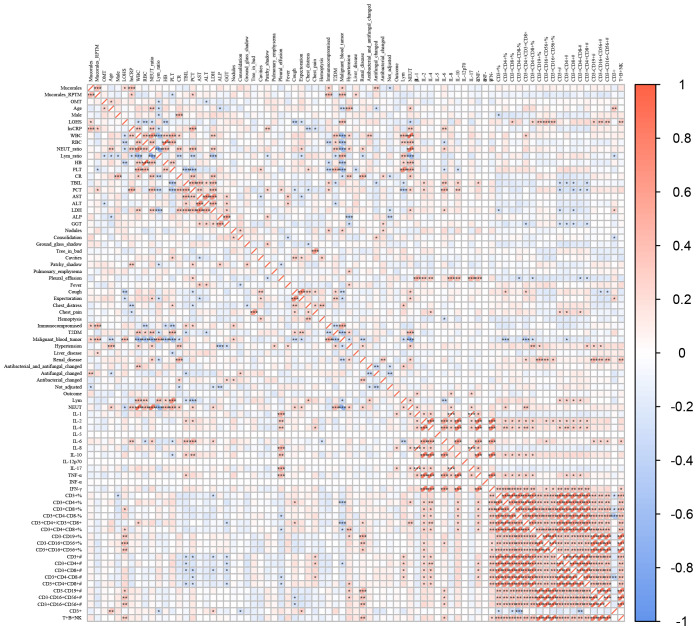
Correlations between the characteristics and *Mucorales* infection. Spearman correlations analysis between *Mucorales* infection and characteristics of patients. *P < 0.05, **P < 0.01, ***P < 0.001.

We conducted Spearman correlation analyses to examine the relationship between various characteristics and *Mucorales* infection. The results showed significant positive correlations between *Mucorales* infection and the following variables: *Mucorales* RPTM value, LOHS, hsCRP, immunocompromised, malignant blood tumor, and antifungal changed. Significant negative correlations between *Mucorales* infection and not adjust drug level were observed. Additionally, significant positive correlations were observed between *Mucorales* RPTM value and the following variables: hsCRP, PCT, immunocompromised, malignant blood tumor, and liver disease. Notably, OMT *Mucorales* positivity was positively correlated with age, and CD3+ index; and negatively correlated with lymphocyte ratio and Alanine Aminotransferase (ALT). Furthermore, positive correlations were found between LOHS and the following variants: B cells, NK cells and NKT cells. Pleural effusion was significantly positive with IL-1, IL-2, IL-4, IL-6, IL-8, IL-17, and TNF-α (**Figure 5**).

### 3.6 Differences in the microbial community structure

**Figure 6:**
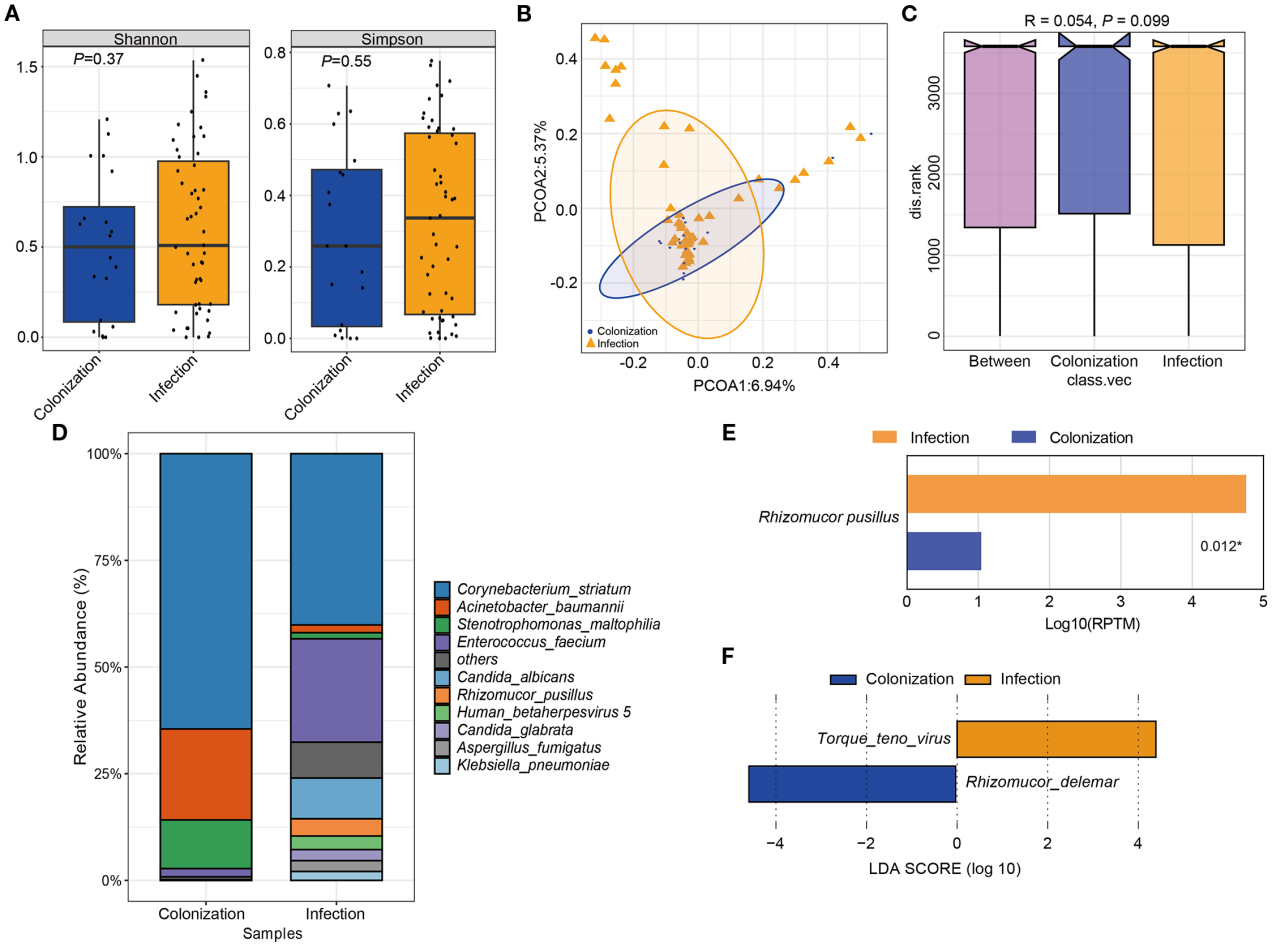
The difference of microbial composition for patients with *Mucorales* infection and colonization. **(A)** Alpha diversity was showed by Shannon and Simpson index. **(B)** PCoA analysis of the microbial composition. **(C)** ANOSIM for the analysis of microbial community structure. **(D)** Barplot showed the top 10 species with the highest abundance between two groups. **(E)** Significant different analysis of the species between two groups with Kruskal-Wallis test. **(F)** Lefse analysis for enriched species for the two groups. *P* < 0.05.

The study compared the overall composition and diversity of the microbial signature in patients with *Mucorales* infection and colonization. Although no significant difference was observed, patients with *Mucorales* infection showed a higher diversity according to both the Shannon and Simpson indices, indicating a trend towards increased richness and evenness of microbial composition (**Figure 6A**). PCoA results indicated that the samples from both groups were intermixed. However, the infection group displayed a wider spread of data compared to the colonization group (**Figure 6B**). Moreover, no significant difference in the microbial community structure between the two groups was observed (**Figure 6C**).

The relative abundance of the top 10 species were *Corynebacterium striatum*, *Acinetobacter baumannii*, *Stenotrophomonas maltophilia*, *Enterococcus faecium*, *Candida albicans*, *Rhizomucor pusillus*, *Human betaherpesvirus 5*, *Candida glabrata*, *Aspergillus fumigatus*, and *Klebsiella pneumoniae*. Among them, only *Rhizomucor pusillus* showed significant differences between the two groups (**Figures 6D, E**). Additionally, two species with LDA scores ≥ 2 and *P* < 0.05 were identified. *Torque teno virus* (TTV) was significantly more abundant in *Mucorales* infection group, whereas *Rhizomucor delemar* was more enriched in *Mucorales* colonization group (**Figure 6F**).

## 4 Discussion

Mucormycosis, a disease with high morbidity and mortality rate, is difficult to diagnose and treat (Cornely et al., 2019). Although *Mucorales* infection and colonization have clear definitions (Cornely et al., 2014a, 2014b; Donnelly et al., 2020), timely and precise diagnosis of invasive *Mucorales* infection or colonization is still complicated and difficult in clinic. However, studies focus on distinguishing *Mucorales* infection from colonization are barely reported. This study was carried out to evaluate the efficacy of mNGS in differentiating *Mucorales* infection from colonization. Moreover, it also outlined the distribution characteristics of mucormycosis, clinical characteristics, immune changes, outcome, antibiotic adjustment of *Mucorales* infection and colonization patients, as well as the variations in sample microbiota.

The main reported pathogens in mucormycosis are *Rhizopus*, *Mucor*, and *Lichtheimia*, followed by *Rhizomucor*, *Cunninghamella*, *Apophysomyces*, and *Saksenaea* (Cornely et al., 2019, 2014a). Consistent with previous researches, our study identified 14 *Mucorales* species among the patients, with 13 species leading to *Mucorales* infection. Among them, *Rhizopus microsporus*, *Rhizopus arrhizus*, and *Rhizomucor pusillus* were the most prevalent in patients with *Mucorales* infection. *Rhizopus delemar*, while *Rhizopus arrhizus*, and *Rhizomucor pusillus* were the most common species in patients with *Mucorales* colonization (**Figure 1**). Additionally, from **Table 1** and spearson correlation analyses, we found that the *Mucorales* RPTM value, LOHS, hsCRP, immunocompromised, malignant blood tumor, and antifungal changed accounted for the *Mucorales* infection, which may be beyond the existed research findings (Cornely et al., 2019).

With the widespread application of mNGS, it offers a hypothesis-free, unbiased approach to pathogen detection, enabling the identification of novel or unexpected organisms, semi-quantitative analysis, and comprehensive genomic coverage. However, its limitations include high host background noise, substantial cost and turnaround time, incomplete reference databases, and susceptibility to environmental contamination (Gu et al., 2019). However, the benefits of using mNGS for pathogen detection have become increasingly apparent, especially for rare and emerging pathogens, such as mucormycosis, hyalohyphomycosis (*Fusarium*, *Paecilomyces*, *Scedosporium*, etc.), and phaeohyphomycosis (*Alternaria*, *Bipolaris*, *Cladosporium*, *Rhinocladiella*, etc.) (Ling et al., 2024; Xing et al., 2023; Wang et al., 2024; Li et al., 2024). A research has shown that mNGS of infected body fluids by Illumina sequencing has a combined sensitivity and specificity of 79% (95% CI 73.5–85.2%) and 91% (95% CI 87.3–93.8%) for bacteria and 91% (95% CI 84.2–100%) and 89% (95% CI 85.7–92.5%) for fungi, respectively (Gu et al., 2021). The above indicates that mNGS is a highly effective option even before OMT results are available. Early and precise detection of pathogen of severe or rare infectious patients is critical for clinicians to give a timely fast intervention and targeted therapy as quickly as possible. It suggests that the medical related organisms including *Candida*, *Cryptococcus*, *Mucorales*, and *Aspergillus* has increased in subjects with impaired immune function, and the thick cell wall of fungi is difficult to break to release nucleic acid which lead to false negative mNGS results (Bittinger et al., 2014). While the diagnostic performance of mNGS has improved with optimized extraction methods (Gu et al., 2021). Besides, the positive diagnostic threshold criteria for mNGS should be defined according to different host and pathogen status. Based on these, this study laid the foundation for the establishment of the positive threshold criteria according to different host and pathogen status in some ways.

Numerous studies have investigated the diagnostic ability of mNGS for *Mucorales* infection, but there remains little research on the distinction of *Mucorales* colonization and infection (Wang et al., 2024; Zhang et al., 2024). Meaningfully, our study laid the foundation for the establishment of the positive threshold criteria according to different host and pathogen status in some ways. We observed that mNGS displayed superior accuracy in diagnosing *Mucorales* infection and distinguishing it from colonization when compared to culture and OMT (*P* < 0.05). The optimal cut-off value of RPTM for mNGS was 51. At this threshold, mNGS achieved a sensitivity of 58.82% and a specificity of 90.00% for the final diagnosis (**Figure 2B**). Furthermore, multiple (≥10) nodules, pleural effusion and halo sign were reportedly associated with pulmonary mucormycosis (Chamilos et al., 2005; Legouge et al., 2014). However, we found that imaging has limitations in diagnosing mucormycosis in clinical, especially when it comes to co-infection of multiple pathogens. Indeed, this research can serve as a valuable reference for analyzing patients with *Mucorales* infection and colonization. Notably, even though mNGS serves as a precise pathogen infection test method and has potential diagnosis in clinic, the final diagnosis of the disease counts on clinical experts who integrate the patient’s symptoms, clinical laboratory test results, and etiological findings to make a comprehensive decision. And in the future, it is necessary for us to conduct prospective studies with a large amount of data about distinction of *Mucorales* infection and colonization.

mNGS had significant impact on treatment regimens, particularly in infectious disease (Zhang et al., 2024). Equally, in this study, 68.18%% and 70.00%% showed improvement among the patients who received only antifungal treatment, and antibacterial combined with antifungal treatment, respectively. This suggested that timely clinical intervention and targeted antifungal therapy for patient prognosis is of great importance. Although Shannon and Simpson indexes were higher in the infection group, no significant differences were observed in species abundance and diversity between the two groups (**Figure 6**). Even the microbial diversity differences are minimal and not statistically significant, these microbiome findings are as exploratory and mainly hypothesis generating. Incidentally, *Rhizomucor pusillus* appeared more frequently in *Mucorales* infection group. Additionally, TTV, and *Rhizomucor delema*r were significantly more abundant in patients with *Mucorales* infection and colonization individually. TTV is a member of *Anellovirida*, which is commonly present in patients with various blood diseases, organ transplants, tumors, periodontitis, and even the healthy population (Maggi and Bendinelli, 2010; Nishizawa et al., 1997). In our study, nine patients were diagnosed with TTV infection, with five patients immunocompromised and three patients suffered from blood disease. However, whether the value of TTV in the infected group indeed existed or was influenced by confounding factors like patients’ immune status, further prospective clinical studies are needed to verify. And further exploration is necessary to deeply understand the potential interaction mechanism between TTV, *Rhizomucor delemar* and *Mucorales* infection. The disparity of the different results of microbiome analysis may because of the advanced age of our patients, their relatively lower mortality rate, their immune status, and no restrictions on the type of diseases they exhibited.

In this study, we conducted a comprehensively retrospective study to analyze the clinical characteristics, immune changes, outcome, antibacterial and antifungal adjustment, and microbiota changes in individuals with *Mucorales* infection and colonization. Furthermore, the efficacy of mNGS was evaluated to distinguish *Mucorales* infection and colonization. With meticulously designed and analyzed, the study also exists limitations. First, not all patients underwent all clinically laboratory tests, which results in a lack of corresponding comparative diagnostic performance results. The second problem relates to the single-center study. Finally, the sample size is indeed small, and the number of some sample types like CSF, pleural fluid, pus, etc. is little, which may cause a bias in the analysis outcomes.

## 5 Conclusions

In this investigation, the performance of mNGS in distinguishing *Mucorales* infection from colonization, with the differences in patients’ clinical characteristics, antibacterial and antifungal adjustment, and microbiota analysis, were analyzed. We found that mNGS has a high diagnostic efficacy for distinguishing *Mucorales* infection and colonization, which was better than culture and OMT used in this retrospective research. Moreover, mNGS played a more important role on the guidance of medication in patients with *Mucorales* infection. Malignant blood tumor, immunocompromised, LOHS, and hsCRP were significant different indicators between patients with *Mucorales* infection from colonization.

## Data Availability

The original contributions presented in the study are included in the article/**Supplementary Material**. Further inquiries can be directed to the corresponding authors.
